# Ultrasound radiomics models based on multimodal imaging feature fusion of papillary thyroid carcinoma for predicting central lymph node metastasis

**DOI:** 10.3389/fonc.2023.1261080

**Published:** 2023-10-30

**Authors:** Quan Dai, Yi Tao, Dongmei Liu, Chen Zhao, Dong Sui, Jinshun Xu, Tiefeng Shi, Xiaoping Leng, Man Lu

**Affiliations:** ^1^ Department of Ultrasound, Sichuan Clinical Research Center for Cancer, Sichuan Cancer Hospital & Institute, Sichuan Cancer Center, Affiliated Cancer Hospital of University of Electronic Science and Technology of China, Medicine & Laboratory of Translational Research in Ultrasound Theranostics, Chengdu, China; ^2^ Department of Ultrasound, West China Hospital of Sichuan University, Chengdu, China; ^3^ Department of Ultrasound, The Second Affiliated Hospital of Harbin Medical University, Harbin, Heilongjiang, China; ^4^ State Key Laboratory of Virtual Reality Technology and Systems, Beihang University, Beijing, China; ^5^ School of Electrical and Information Engineering, Beijing University of Civil Engineering and Architecture, Beijing, China; ^6^ Department of General Surgery, The Second Affiliated Hospital of Harbin Medical University, Harbin, Heilongjiang, China

**Keywords:** lymph node metastasis, multimodality, papillary thyroid carcinoma, radiomics, ultrasound

## Abstract

**Objective:**

This retrospective study aimed to establish ultrasound radiomics models to predict central lymph node metastasis (CLNM) based on preoperative multimodal ultrasound imaging features fusion of primary papillary thyroid carcinoma (PTC).

**Methods:**

In total, 498 cases of unifocal PTC were randomly divided into two sets which comprised 348 cases (training set) and 150 cases (validition set). In addition, the testing set contained 120 cases of PTC at different times. Post-operative histopathology was the gold standard for CLNM. The following steps were used to build models: the regions of interest were segmented in PTC ultrasound images, multimodal ultrasound image features were then extracted by the deep learning residual neural network with 50-layer network, followed by feature selection and fusion; subsequently, classification was performed using three classical classifiers—adaptive boosting (AB), linear discriminant analysis (LDA), and support vector machine (SVM). The performances of the unimodal models (Unimodal-AB, Unimodal-LDA, and Unimodal-SVM) and the multimodal models (Multimodal-AB, Multimodal-LDA, and Multimodal-SVM) were evaluated and compared.

**Results:**

The Multimodal-SVM model achieved the best predictive performance than the other models (*P* < 0.05). For the Multimodal-SVM model validation and testing sets, the areas under the receiver operating characteristic curves (AUCs) were 0.910 (95% CI, 0.894-0.926) and 0.851 (95% CI, 0.833-0.869), respectively. The AUCs of the Multimodal-SVM model were 0.920 (95% CI, 0.881-0.959) in the cN0 subgroup-1 cases and 0.828 (95% CI, 0.769-0.887) in the cN0 subgroup-2 cases.

**Conclusion:**

The ultrasound radiomics model only based on the PTC multimodal ultrasound image have high clinical value in predicting CLNM and can provide a reference for treatment decisions.

## Introduction

The incidence of thyroid cancer continues to increase, with the main pathological type being papillary thyroid carcinoma (PTC), which accounts for 80–90% of diagnosed cases (1). An important risk factor affecting survival and recurrence of PTC is cervical lymph node metastasis (LNM) (2). The central region of the neck is usually the most dangerous and the earliest to metastasize (3). Previous studies had showed that approximately 30–80% of cases with PTC were associated with central lymph node metastasis (CLNM) (4). In view of the high-clinical risk associated with positive lymph nodes in PTC patients, some researchers have recommended routine central lymph node dissection (CLND) in the initial surgery to improve treatment outcomes (5). However, it is still debated whether prophylactic CLND is beneficial in the case of well-differentiated PTC. Prophylactic CLND has been associated with an increased risk of postoperative complications, such as transient and permanent hypoparathyroidism, unexpected recurrent laryngeal nerve, and peripheral vascular injury (6, 7). During the PTC therapy, the presence or absence of CLNM could affect the surgical approach and postoperative staging. A standardized surgical approach and cervical lymph node dissection strategy may increase the curative rate of the disease and reduce the rate of complications. Therefore, accurate assessment of CLNM noninvasively before surgery is critical as it helps clinicians develop surgical plans and assess prognosis.

Ultrasound is extensively used as the imaging method for thyroid and cervical lymph nodes (8, 9). However, owing to the special anatomy of the central region of the neck, the direct preoperative detection ability of ultrasound for CLNM is limited, with a sensitivity in the range of 15–40% (10, 11). Therefore, there is an urgent need for more accurate and efficient methods to assess the risk of CLNM in PTC patients. Because the characteristics of the primary tumor are closely related to its invasiveness and metastasis, the cervical lymph node status can be assessed further by analyzing the grayscale, color Doppler flow imaging (CDFI), and strain elastography ultrasound characteristics of the primary PTC (12, 13). In recent years, radiomics has been used in clinical practice for preoperative prediction and prognostic assessments of diseases. Researchers have developed models based on traditional radiomics features (such as intensity, texture, and wavelet features) of grayscale or elastography images of primary PTC to predict CLNM. The areas under curves (AUCs) range in the validation set was found to be in the range of 0.727–0.858 (14–16), these outcomes suggest a new research direction for preoperative assessment of CLNM.

With the continuous development and application of artificial intelligence in the medical field, deep-learning algorithms have attracted widespread attention because of their excellent performance in image recognition tasks and have proven to be useful in medical imaging (17, 18). Deep-learning algorithms have been used extensively for medical image diagnosis and prediction owing to their speed, accuracy, and reproducibility advantages (19). Additionally, studies have indicated that the accuracy and reliability of traditional radiomics for medical image classification or prediction can be improved with the introduction of deep-learning algorithms (20). Grayscale ultrasound of PTC reveals the lesion characteristics from a morphological viewpoint, CDFI reveals the characteristics of the blood flow distribution in the lesion area, and elastography provides information on the relative stiffness of the lesion. These imaging tests can be implemented as noninvasive ultrasound examinations (21), and the combined application of these tests may provide more imaging information and indirectly reflect more characteristics associated with tumor growth, invasiveness, and metastasis. To our knowledge, the combination of multimodal ultrasound features of primary PTC tumors and their use to develop a radiomics model to predict CLNM has not been reported.

Therefore, this study aimed to develop a prediction model using multimodal ultrasound image feature fusion based on radiomics and to preoperatively evaluate the risk of CLNM in PTC patients.

## Materials and methods

### Patients and datasets

This retrospective study was approved by the Ethics Committee of The Second Affiliated Hospital of Harbin Medical University, and the requirement for informed patient consent given the study’s nature was waived (approval number: KY2021-152).

We included patients (1) with a postoperative pathological diagnosis of PTC (2), who underwent thyroid surgery with CLND for the first time (3), whose preoperative intrathyroid lesions were not treated using other methods, such as induction chemotherapy or neck radiation therapy (4), whose thyroid ultrasound examination was performed in our institution within 1 week before surgery, and (5) cases wherein the ultrasound imaging of the primary tumor was visible. The exclusion criteria were as follows (1): distant metastasis or other malignant tumors (2), skip metastasis (3), incomplete ultrasound imaging information or the suspected tumor lesions on ultrasound were inconsistent with the pathological results (4), undetected thyroid tumors by ultrasound, and (5) incomplete medical records.

**Figure 1:**
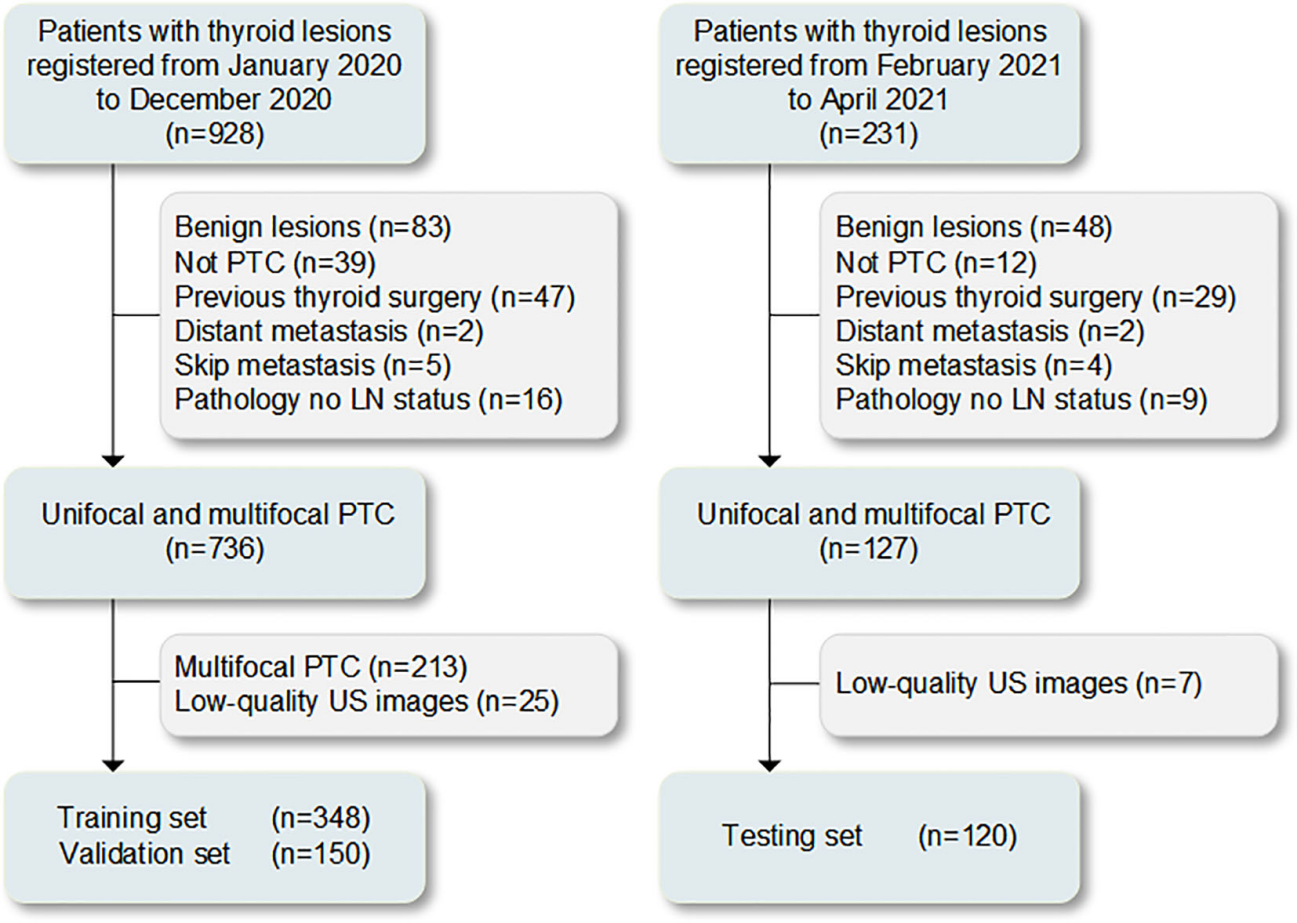
Process of patient enrollment for the study. LN, lymph node; PTC, papillary thyroid carcinoma; US, ultrasound.

Overall, 928 cases with resectable tumors were comprehensive surgical treatment in The Second Affiliated Hospital of Harbin Medical University from January 2020 to December 2020. In total, 498 patients with unifocal PTC for the first operation conforming to the inclusion criteria were selected (**Supplementary Text 1**). The Scikit-learning frame (Python, version 3.6.8) was used to divided the patients into the training (n=348) and validation (n=150) sets at a ratio of 7:3 randomly. Subsequently, 231 cases with resectable tumors were comprehensive surgical treatment in the same hospital from February 2021 to April 2021. Among 120 patients with unifocal or multifocal PTC who were screened with the same criteria used for the testing set (**Supplementary Text 1**). The procedures for the enrollment of patients are shown in **Figure 1**. The cervical lymph node status was determined based on the postoperative pathological results (CLNM-positive and CLNM-negative). Clinical data were obtained from medical records. The clinical characteristics of the patients are presented in **Table 1**.

**Table 1:** Patients characteristics of the three datasets.

Characteristics	Training set			Validation set			Testing set		
	CLNM (+) (n=167)	CLNM (–) (n=181)	*P value*	CLNM (+) (n=74)	CLNM (–) (n=76)	*P value*	CLNM (+) (n=58)	CLNM (–) (n=62)	*P value*
Age, years									
range	21~68	22~69		21~57	24~70		24~69	22~66	
Mean ± SD	42.4 ± 10.7	46.9 ± 9.3	<0.001	40.8 ± 9.0	46.3 ± 8.8	<0.001	41.7 ± 11.0	46.4 ± 8.7	0.012
<45	101(60.5)	68(37.6)	<0.001	44(59.5)	28(36.8)	0.006	36(62.1)	22(35.5)	0.004
≥45	66(39.5)	113(62.4)		30(40.5)	48(63.2)		22(37.9)	40(64.5)	
Gender, n (%)									
Male	47(28.1)	27(14.9)	0.003	17(23.0)	12(15.8)	0.265	16(27.6)	8(12.9)	0.044
Female	120(71.9)	154(85.1)		57(77.0)	64(84.2)		42(72.4)	54(87.1)	
US tumor size, mm									
range	3.7~41.6	2.7~30.6		3.7~53.4	4.2~19.9		4.9~28.2	3.2~25.7	
Mean ± SD	12.0 ± 6.7	7.9 ± 4.2	<0.001	13.2 ± 8.8	7.7 ± 3.0	<0.001	13.5 ± 6.1	9.9 ± 5.1	<0.001
≤10mm	85(50.9)	149(82.3)	<0.001	39(52.7)	66(86.8)	<0.001	20(34.5)	44(71.0)	<0.001
>10mm	82(49.1)	32(17.7)		35(47.3)	10(13.2)		38(65.5)	18(29.0)	
US reported LN status, n (%)									
Suspicious	61(36.5)	7(3.9)	<0.001	32(43.2)	2(2.6)	<0.001	16(27.6)	4(6.5)	0.003
No suspicious	106(63.5)	174(96.1)		42(56.8)	74(97.4)		42(72.4)	58(93.5)	

Categorical variables were described as the number of patients (percentages are listed in parentheses).

Continuous variables are presented as mean ± standard deviation (SD). The P-value indicates whether a significant difference exists between the CLNM (+) and CLNM (–). US, ultrasound; LN, lymph node.

### Ultrasound image acquisition

Preoperative ultrasound images were collected by two board-certified sonographers (Q.D. and Y.T., with 15 and 4 years of experience, respectively) using a HITACHI HIVISION Avius (Hitachi Medical Corporation) equipped with a 5–13MHz linear probe. Based on the thyroid scanning method and imaging parameter adjustment requirements, ultrasound images were adjusted to achieve optimal thyroid-imaging effects (9). Transverse and longitudinal grayscale, CDFI, and strain elastography static images showing the maximum diameter of the lesion were acquired and stored twice for all unifocal cases, with each image containing as many typical malignant features as possible (**Supplementary Text 2**). The ultrasound image of tumor with the largest volume among multifocal cases were acquired and stored twice according to the above procedure. The dynamic images of the complete thyroid were acquired and stored in all PTC cases at the same time. The Kappa consistency test was performed on the image quality of the same sonographer. Finally, a total of 3708 high-quality static images were selected and converted to the portable network graphic format for deep-learning feature extraction with an image resolution of 1024 × 768. The tumor size was defined as the maximum diameter measured by ultrasound in the transverse or longitudinal cross-section. Ultrasound reports the cervical lymph nodes status according to other relevant studies (9, 22).

### Segmentation of regions of interest and feature extraction

**Figure 2:**
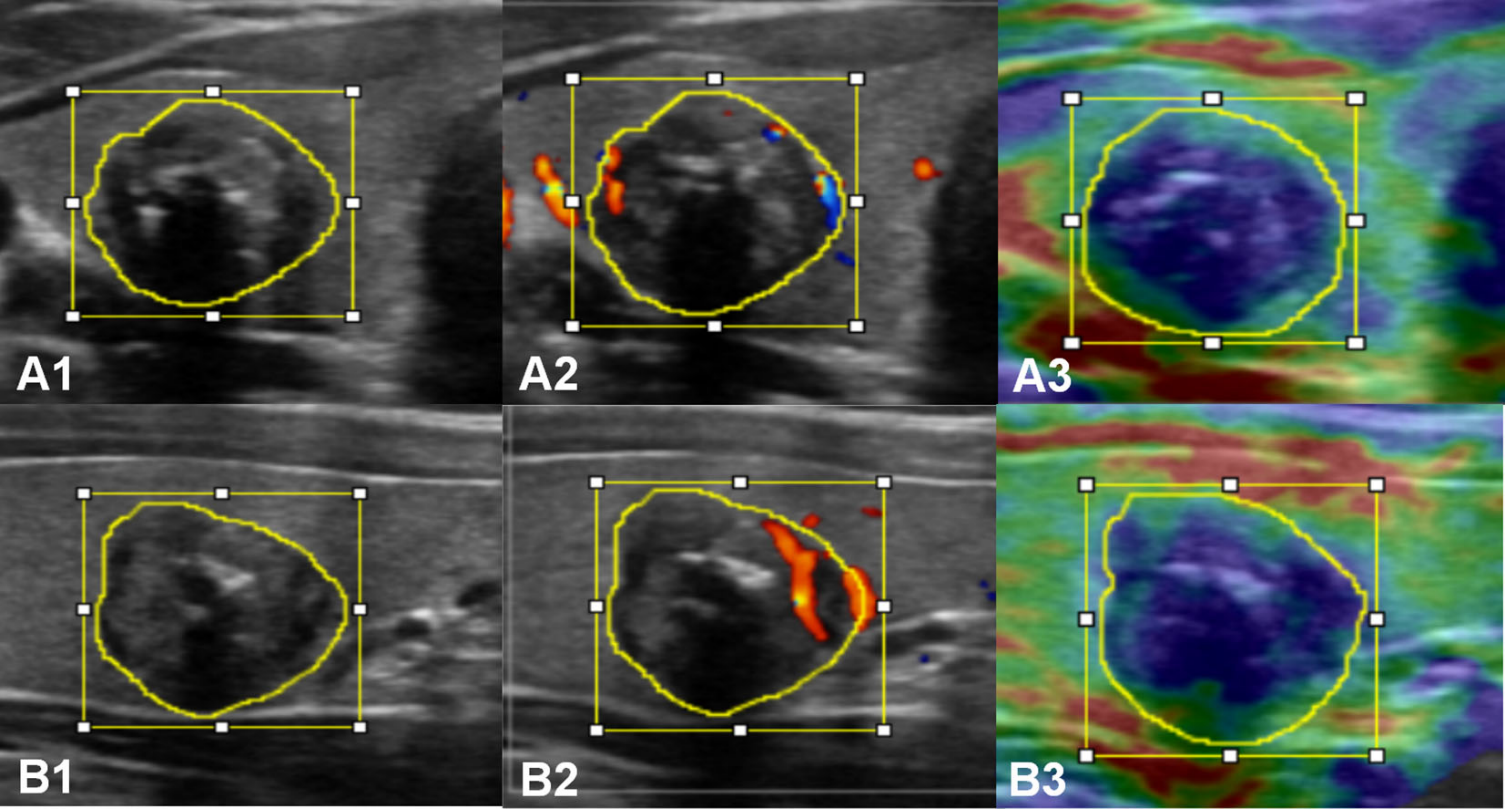
Multimodal ultrasound image ROI annotation of a primary PTC in the transverse **(A)** and longitudinal **(B)** sections. A1 and B1, grayscale; A2 and B2, CDFI; A3 and B3, strain elastography.

The lesion regions were manually segmented by two sonographers (Q.D. and D.L., with more than 10 years of experience, respectively), and regions of interest (ROI) annotation was performed on the lesion regions of each section image for each modality using ImageJ software (version 1.48, National Institutes of Health, USA). The Kappa consistency test was performed on the consistency of the static images selected by the two sonographers and the satisfaction of the ROI annotation. The ROI refers to the smallest rectangular box that contained the boundaries of the tumor (i.e., the bounding box) and the area by tracing along the edge of the lesion (i.e., the mask) in each image (**Supplementary Text 3**). An example of multimodal ultrasound image ROI annotation of primary PTC is shown in **Figure 2**.

To avoid losing potential feature information, no preprocessing, such as enhancement or noise reduction, was performed on the raw images before data analysis. In addition, because information such as blood vessels around the lesion, image markers, and annotations may have interfered with the model training and led to incorrect learning, only the masked region was used as the input data in this study.

**Figure 3:**
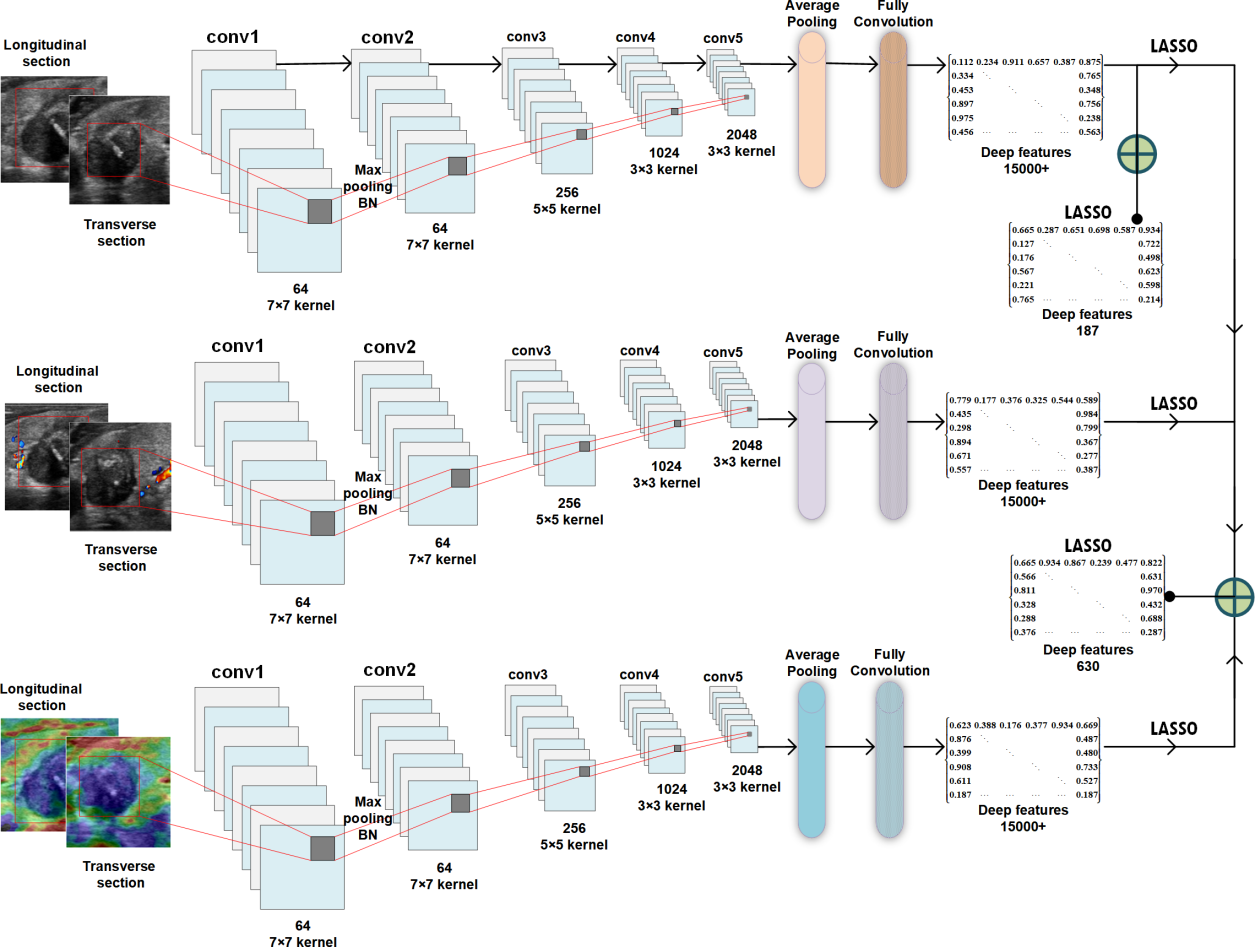
Schematic of deep feature extraction, selection, and fusion for the ResNet50 network.

For image feature extraction, the primary tumor regions annotated with different modal image ROI (i.e., the mask) were used as input images, and the pretrained deep convolutional neural network (CNN) model of the residual neural network with 50-layer (ResNet50) network architecture was used for feature extraction (**Figure 3**). The CNN is a well-known and popular type of deep-learning architecture, which can also learn useful texture features automatically for classification, thus producing results superior to those of other methods (23). ResNet50 (Keras Applications, https://keras.io/api/applications/) is one of the best-performing model architectures for representation, which has proven to be competitive for differential diagnosis using various medical images (24, 25). In the present study, during the model training process, the masked regions of the primary tumor were input in the pretrained ResNet50 network (image feature extraction framework) and were connected to the subsequent image classification task. The ResNet50 network was pretrained using the ImageNet (http://www.imagenet.org/) dataset with default network parameter settings. This network is capable of extracting multiscale and multidimensional image features for operational analysis. In this study, the ResNet50 network consisted of five convolutional layers, six pooling layers, and one fully connected layer. The network respectively extracted high-dimensional features from the multimodal ultrasound images at different scales and network layers for the final classification task; more than 15000 deep features were extracted from each image (**Figure 3**).

### Feature selection and fusion

After the image features were extracted, dimensionality reduction was performed using the least absolute shrinkage and selection operator (LASSO) algorithm (26). Various features were retained according to the correlations among the modal features. Subsequently, the features were selected for the final classification task according to two principles (1): the elimination of high-dimensional features extracted from the network (e.g., morphological and contour features) and (2) the balancing of network features of different dimensions to ensure that the model achieved high accuracy for classification with minimal overfitting and underfitting.

In the feature fusion phase, the grayscale image features of the transverse and longitudinal sections were fused to build the unimodal model; all the features of the grayscale, CDFI, and strain elastography images of the transverse and longitudinal sections were fused to build the multimodal model. The feature fusion methods of each modal for the prediction model are shown in **Figure 3**. All the fusion methods are based on traditional early fusion; this is a feature-level fusion technique performed at the stage of feature appearance that directly combines features extracted from unimodal data of different cross-sections to emphasize intra-model interactions while suppressing inter-model interactions (27).

### Classification and model construction

**Figure 4:**
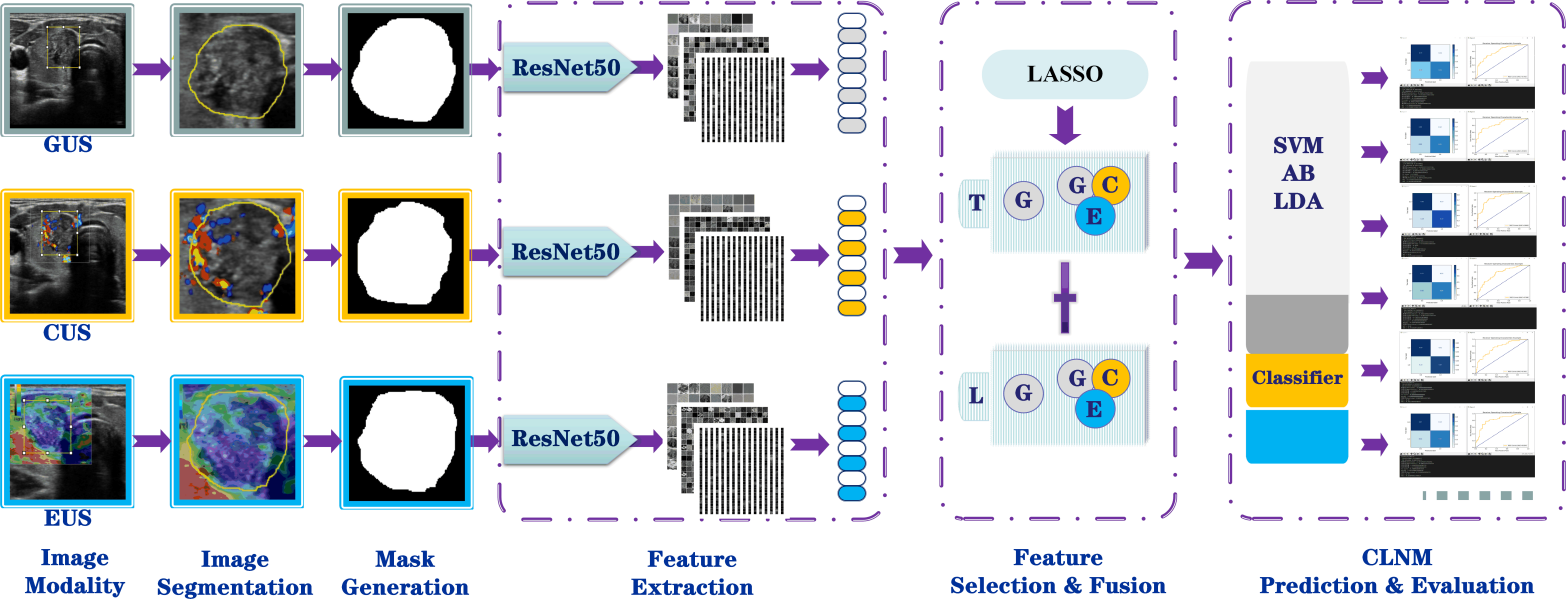
Workflow for building the radiomics models based on multimodal US image feature fusion. G, grayscale; C, CDFI; E, elastography; US, ultrasound; T, transverse section; L, longitudinal sections; AB, adaptive boosting; LDA, linear discriminant analysis; SVM, support vector machine.

In the model classification and prediction phase, the unimodal and multimodal feature fusion data are respectively applied to three types of classifiers classify, i.e., adaptive boosting (AB), linear discriminant analysis (LDA), and support vector machine (SVM), completing the model building. A brief description of these classifiers is shown in **Supplementary Text 4**. The process of constructing the models is shown in **Figure 4**. In this study, a total of six CLNM prediction models were obtained: Unimodal-AB, Unimodal-LDA, Unimodal-SVM, Multimodal-AB, Multimodal-LDA, and Multimodal-SVM. To avoid overfitting and underfitting, threefold cross-validation was used to train and test these models according to the sample size of the available dataset to ensure that the maximum amount of information was obtained from the limited data and to build a more robust model.

### Model evaluations and statistical analysis

The six trained prediction models were tested in the validation and testing sets and the model with the best performance was used to perform evaluations for the cN0-stage subgroup cases. For each dataset, the models’ CLNM classification results of 0 or 1 were used as the predicted outcomes [0 indicated CLNM (–) and 1 indicated CLNM (+)]. The predicted results of each model were compared with the actual lymph node status in the pathology report [CLNM (–) and CLNM (+) were marked as 0 and 1, respectively]. All the code for the model construction and data analysis was stored on GitHub (ID: https://github.com/daixiaoxiao520/Multi-module). Data operations were performed on a computer that operated 64-bit Windows 10 with a Nvidia 2080 Ti graphics card and 11.6 GB of video memory.

The software programs Python (version 3.6.8), SPSS version 25.0 (IBM, Armonk, New York, USA.), and Medcalc version 19.4.0 were used for data processing and statistical analysis. Categorical and continuous variables are expressed as frequencies and percentages and as mean ± standard deviation, respectively. Chi-squared or Fisher’s exact tests were used for rate comparisons. Independent samples t and Mann–Whitney U tests were used for mean value comparisons. Inter- and intra-observer agreement was evaluated using Kappa consistency test. Comparisons between AUCs were made by using the DeLong’ test (28). The criterion for statistically significant differences was *P* < 0.05.

## Results

### Clinical and pathological data

In the training, validation and testing sets, the positive CLNM rates were 48.0% (167/348), 49.3% (74/150) and 48.3% (58/120), respectively. There was no significant difference in the positive rate among the three sets (*P*=0.963). **Table 1** briefly compared baseline data, such as age, gender, tumor size by preoperative ultrasound, and lymph-node status of central or/and lateral neck by preoperative ultrasound. In this study, 38.3% (190/496) of cN0 staging PTC cases were pathologically confirmed CLNM (+). The weighted Kappa consistency indicated a high degree of intra- and inter-observer agreement (range, 0.744-0.946) (**Supplementary Tables 1**, **2**).

### Performance of ultrasound radiomics model

**Figure 5:**
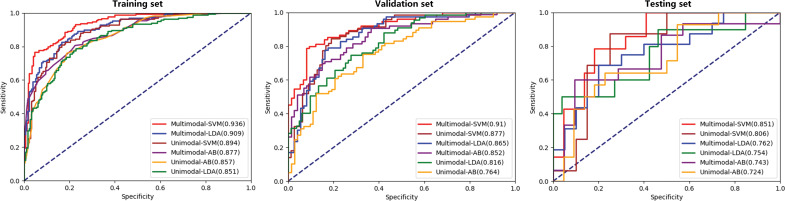
ROC curves of the six ultrasound radiomics models in the three datasets.

In this study, the AUC, accuracy, sensitivity, specificity, positive predictive value (PPV), negative predictive value (NPV), F1-score, recall and precision values from the six models are shown in **Table 2**. The receiver operating characteristic (ROC) curves of each model in the training, validation and testing sets are shown in **Figure 5**. In the three unimodal models, Unimodal-SVM had the best performance and the AUCs were 0.877 and 0.806 in the validation and testing sets, respectively. The accuracy, sensitivity, specificity, and precision of the Unimodal-SVM were 0.820, 0.831, 0.810, and 0.814 in the validation set and 0.722, 0.875, 0.600 and 0.686 in the testing set, respectively. In the three multimodal models, Multimodal-SVM yielded the best performance and the AUCs were 0.910 and 0.851 in the validation and testing sets, respectively. The accuracy, sensitivity, specificity, and precision of the Multimodal-SVM were 0.847, 0.800, 0.893, and 0.882 in the validation set and 0.750, 0.857, 0.682 and 0.729 in the testing set, respectively. Multimodal-SVM outperformed the other five models in the validation and testing sets according to the DeLong’s test (*P* < 0.05), with higher AUC, accuracy, and precision values. The classification confusion matrices that report the ratio of true-positive, false-positive, true-negative, and false-negative results for the ultrasound radiomics models are shown in **Table 3**.

**Table 2:** Performance of the ultrasound radiomics models for predicting central lymph node metastasis.

Data set	Metrics	Unimodal			Multimodal		
		AB	LDA	SVM	AB	LDA	SVM
**Training** **set**	**AUC** **(95%CI)**	0.857* (0.830-0.884)	0.851* (0.827-0.875)	0.894* (0.872-0.916)	0.877* (0.853-0.901)	0.909* (0.893-0.925)	**0.936** (0.920-0.952)
	**Accuracy**	0.787	0.773	0.816	0.790	0.828	**0.842**
	**Sensitivity**	0.759	0.774	0.808	0.806	0.807	**0.823**
	**Specificity**	0.812	0.772	0.823	0.775	0.846	**0.861**
	**NPV**	0.771	0.774	0.811	0.800	0.814	**0.829**
	**PPV**	0.801	0.772	0.821	0.782	0.840	**0.856**
	**F1-score**	0.769	0.763	0.808	0.790	0.817	**0.840**
	**Recall**	0.759	0.774	0.808	0.806	0.807	**0.823**
	**Precision**	0.801	0.772	0.821	0.782	0.840	**0.856**
**Validation** **set**	**AUC** **(95%CI)**	0.764* (0.717-0.811)	0.816* (0.791-0.842)	0.877* (0.838-0.916)	0.852* (0.805-0.899)	0.865* (0.851-0.879)	**0.910** (0.894-0.926)
	**Accuracy**	0.680	0.720	0.820	0.747	0.807	**0.847**
	**Sensitivity**	0.688	0.687	**0.831**	0.724	0.776	0.800
	**Specificity**	0.671	0.747	0.810	0.770	0.838	**0.893**
	**NPV**	0.683	0.704	**0.827**	0.736	0.789	0.817
	**PPV**	0.677	0.731	0.814	0.759	0.827	**0.882**
	**F1-score**	0.688	0.687	0.814	0.743	0.803	**0.839**
	**Recall**	0.688	0.687	**0.831**	0.724	0.776	0.800
	**Precision**	0.677	0.731	0.814	0.759	0.827	**0.882**
**Testing** **set**	**AUC** **(95%CI)**	0.724* (0.704-0.744)	0.754* (0.732-0.776)	0.806* (0.784-0.828)	0.743* (0.721-0.765)	0.762* (0.744-0.78)	**0.851** (0.833-0.869)
	**Accuracy**	0.583	0.611	0.722	0.583	0.722	**0.750**
	**Sensitivity**	0.714	0.600	**0.875**	0.667	0.750	0.857
	**Specificity**	0.500	0.615	0.600	0.524	**0.700**	0.682
	**NPV**	0.636	0.606	**0.827**	0.611	0.737	**0.827**
	**PPV**	0.588	0.609	0.686	0.583	0.714	**0.729**
	**F1-score**	0.571	0.462	**0.737**	0.571	0.706	0.727
	**Recall**	0.714	0.600	**0.875**	0.667	0.750	0.857
	**Precision**	0.588	0.609	0.686	0.583	0.714	**0.729**

Bold values indicate the best results. * represents that there is a significant difference existed when multimodal-SVM compared with other five models. AB, adaptive boosting; LDA, linear discriminant analysis; SVM, support vector machine; AUC, areas under curve; 95%CI, 95% confidence interval; NPV, negative predictive value; PPV, positive predictive value.

**Table 3:** Confusion matrices of the models for the different datasets.

Prediction	Unimodal (True)						Multimodal (True)					
	AB		LDA		SVM		AB		LDA		SVM	
	(+)	(–)	(+)	(–)	(+)	(–)	(+)	(–)	(+)	(–)	(+)	(–)
Training set												
**CLNM (+)**	0.76	0.19	0.77	0.23	0.81	0.18	0.81	0.22	0.81	0.15	0.82	0.14
**CLNM** (–)	0.24	0.81	0.23	0.67	0.19	0.82	0.19	0.78	0.19	0.85	0.18	0.86
Validation set												
**CLNM (+)**	0.69	0.33	0.69	0.25	0.83	0.19	0.72	0.23	0.78	0.16	0.80	0.11
**CLNM** (–)	0.31	0.67	0.31	0.75	0.17	0.81	0.28	0.77	0.22	0.84	0.20	0.89
Testing set												
**CLNM (+)**	0.71	0.50	0.60	0.38	0.88	0.40	0.67	0.48	0.75	0.30	0.86	0.32
**CLNM** (–)	0.29	0.50	0.40	0.62	0.12	0.60	0.33	0.52	0.25	0.70	0.14	0.68

### Performance of multimodal-SVM model for cN0-stage tumors

**Figure 6:**
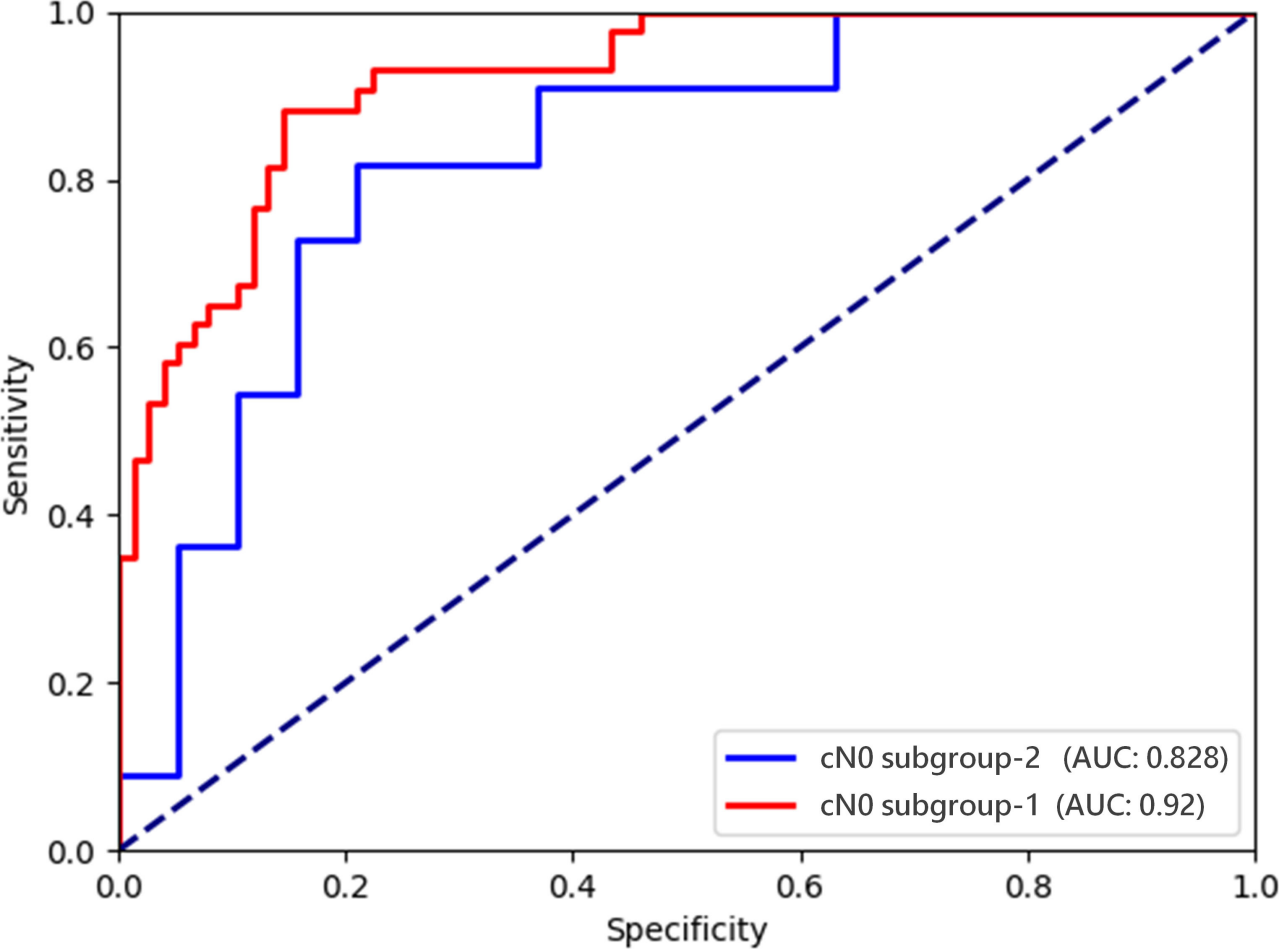
ROC curves of the Multimodal-SVM model for the cN0 subgroups.

**Figure 7:**
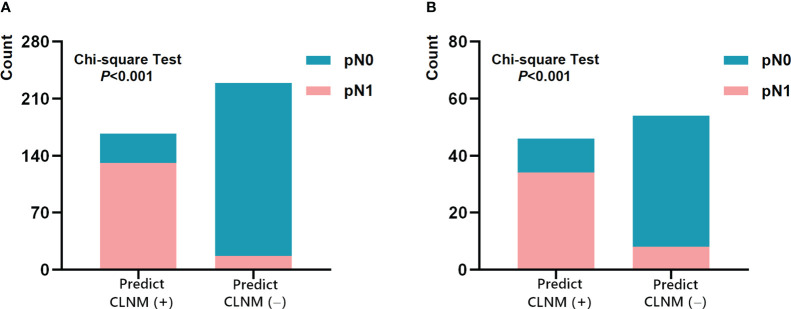
Performance of Multimodal-SVM model in the cN0 subgroup-1 **(A)** and subgroup-2 **(B)**.

The performances of Multimodal-SVM for predicting CLNM in PTC cases in the cN0 subgroup were analyzed. There were 396 patients in the cN0 subgroup-1 from the training and validation sets, and the AUC, accuracy, precision, sensitivity, specificity, NPV, and PPV achieved by the model were 0.920, 0.866, 0.859, 0.884, 0.855, 0.880, and 0.859, respectively. There were 100 patients in the cN0 subgroup-2 from the testing set, and the AUC, accuracy, precision, sensitivity, specificity, NPV, and PPV achieved by the model were 0.828, 0.800, 0.795, 0.818, 0.789, 0.813, and 0.795, respectively, as shown in **Table 4**. The ROC curves of Multimodal-SVM for predicting the presence of CLNM in the cN0-stage subgroup of cases for different datasets are shown in **Figure 6**. The Multimodal-SVM model exhibited good performance for predicting CLNM for the cN0 stage. It is worth noting that the actual CLNM rates are all higher (*P* < 0.001) for patients with predicting CLNM (+) in the both subgroup datasets (**Figure 7**).

**Table 4:** Performance of the Multimodal-SVM model for the cN0 subgroup.

Data sets	AUC (95%CI)	Accuracy	Precision	Sensitivity	Specificity	NPV	PPV
**cN0 subgroup-1**	0.920 (0.881-0.959)	0.866	0.859	0.884	0.855	0.880	0.859
**cN0 subgroup-2**	0.828 (0.769-0.887)	0.800	0.795	0.818	0.789	0.813	0.795

## Discussion

In this study, the grayscale, CDFI and strain elastography ultrasound image features of PTC primary tumors in transverse and longitudinal sections were extracted for feature selection and fusion using the generic ResNet50 network. Classical classifiers (AB, LDA, and SVM) were then used for classification, and six radiomics models for predicting CLNM of PTC were developed. The best-performing model—i.e., Multimodal-SVM—obtained satisfactory predictions for both the validation and testing sets, with AUCs of 0.910 and 0.851, respectively. These results of this study indicated that a modeling approach based on a multimodal ultrasound image feature fusion framework combined with classical classifiers is feasible for predicting CLNM, and the approach can improve the predictive accuracy compared with the unimodal grayscale ultrasound in a limited number of training datasets (14, 16). The PTC patients with absence of any preoperative evidence of lymph node metastasis (cN0 stage) have a high proportion of CLNM confirmed by postoperative pathology. Owing to the low sensitivity associated with the direct detection of CLNM by preoperative ultrasound, the multimodal ultrasound radiomics model can be applied to guide decision making before surgery and as a noninvasive and objective tool for screening the CLN status of PTC.

Studies have indicated that ultrasound image and clinical features of PTC primary tumors are strongly correlated with LNM (29, 30). Although these features may indicate some important information, quantitative and objective assessments are impossible. Meanwhile, because the accuracy varies among sonographers according to the subjective visual and empirical assessment of ultrasound image features, the accuracy of preoperative evaluation of CLNM is directly affected by operator-dependent qualitative analysis. In this study, the Multimodal-SVM model was applied to assess the status of CLN by only using preoperative multimodal (grayscale, CDFI, and strain elastography) ultrasound images of PTC primary tumors. Regardless of whether the suspected CLNs were detected by preoperative ultrasound, the predictive performance of the model was not affected. Only if high-quality preoperative ultrasound images of the primary tumor are acquired, accurate prediction results could be obtained using the proposed framework of deep-learning feature extraction, selection and fusion, and prediction evaluation process, thus confirming the superiority and objectivity of the prediction model and indicating its broad application prospects. The results of this study highlight the effectiveness of artificial intelligence prediction methods based on medical image data processing for clinical diagnosis and treatment.

In this study, the traditional radiomics feature extraction was not applied in the multimodal ultrasound feature extraction stage; instead, the automatically extracted multidimensional features of each modality from the output of the deep-learning CNN network were selected and fused and then input to the traditional classifier for prediction. The model was tested on multimodal ultrasound images; it yielded better results than the unimodal models tested for predicting CLNM. In recent years, deep learning has been extensively employed for medical image classification, and as an important branch of artificial intelligence, it is considered the most advanced image classification technique (31). Previous research reported the advantages of the deep learning algorithm that treats the imaging as a pixel-by-pixel volume in the task of prediction, and this quantitative assessment of imaging information can result in more accurate and reproducible imaging diagnoses than qualitative reasoning (32). Deep-learning algorithms differ from judgments by human vision on medical images in that the algorithm makes the final prediction of the overall image features in different anatomical regions rather than one or more features in the lesion image (33). In contrast to traditional radiomics feature extraction methods, deep learning achieves hierarchical feature extraction (from global to local) and has a greater advantage with regard to the number of features extracted. Compared with several related studies (14–16, 34–37), the combination of deep learning algorithms and shallow machine learning classification algorithm to establish a prediction model is somewhat innovative.

After the model was trained with a large number of manually labeled datasets, the prediction was made according to the image features of the lesion area of the primary tumor of PTC, increasing the amount of information used for evaluation, and maximizing the use of existing image data. This method of constructing prediction models has also been applied to X-ray, computed tomography, and magnetic resonance imaging multimodal images and has achieved good performance for classification and prediction (38, 39). In our study, the AUCs of Multimodal-SVM exceeded those of traditional radiomics models based on grayscale, shear wave elastography and contrast-enhanced ultrasound images for predicting CLNM (AUC: 0.727–0.880) reported in previous studies (14–16, 35). Compared with the previous study, the Multimodal-SVM model showed high precision while maintaining high sensitivity (34). Furthermore, the results were better than those of the traditional radiomics based on computed tomography images for predicting LNM (AUC: 0.709–0.822; accuracy: 0.642–0.670) (40, 41). The model for predicting cases in the cN0-stage subgroup also yielded good performance outcomes, thus indicating the clinical usefulness of the model for assessing the cervical lymph nodes status of PTC.

In this study, the transverse and longitudinal image features of primary tumors were fused for modeling. The multimodal fusion of transverse and longitudinal ultrasound image features provided more scales and diverse high-dimensional image information compared with those from a single section or a unimodal case. Previous studies have indicated that computer-aided diagnosis or radiomics methods combining CDFI or elastography image features can increase the accuracy and sensitivity of disease prediction and classification and can thus improve the diagnostic and predictive performance (42, 43). Our study proved that combining CDFI and strain elastography with grayscale ultrasound images to build a radiomics model with the multimodal ultrasound image features fusion offers better predictive performance than the radiomics model with the unimodal (grayscale) image features fusion.

Several limitations of this study should be noted. First, this study was a single-center retrospective study, and although it proved that the prediction model achieves good predictive performance, future prospective studies are needed to collect relevant data from multiple institutions for further validation to reduce possible biases. Second, obtaining high-quality multimodal images is a prerequisite for using the model; thus, quality control of ultrasound imaging is crucial, and standard operating procedures should not be disregarded. Third, the size of the multimodal datasets should be increased. In future studies, we will continue to introduce images acquired using techniques such as superb microvascular imaging and contrast-enhanced ultrasound, or introduce other imaging and clinical data to increase the diversity of data and obtain more valuable multimodal data for model optimization.

## Conclusion

We demonstrated that an ultrasound radiomics model can predict with high accuracy the presence of CLNM from multimodal ultrasound images of primary PTC. The clinical value of multimodal ultrasound imaging in disease prediction and evaluation is thus improved. This strategy may be an effective approach to early screening for CLNM in clinical lymph node-negative PTC tumors. The multimodal ultrasound radiomics model has great potential in serving as an important decision-support tool in clinical applications.

## Data availability statement

The raw data supporting the conclusions of this article will be made available by the authors, without undue reservation.

## Ethics statement

The studies involving humans were approved by The Ethics Committee of The Second Affiliated Hospital of Harbin Medical University. Written informed consent for participation was not required for this study in accordance with the national legislation and the institutional requirements.

## Author contributions

QD: Conceptualization, Investigation, Writing – original draft, Writing – review & editing, Methodology, Software. YT: Investigation, Writing – original draft, Writing – review & editing. DL: Investigation, Writing – original draft, Formal Analysis, Methodology, Software. CZ: Investigation, Writing – original draft, Formal Analysis. DS: Investigation, Writing – original draft, Methodology, Software. JX: Writing – review & editing. TS: Formal Analysis, Writing – review & editing. XL: Conceptualization, Writing – review & editing, Supervision. ML: Conceptualization, Investigation, Writing – original draft, Writing – review & editing, Supervision.
