# Supplementary material for: Ultrasound radiomics models based on multimodal imaging feature fusion of papillary thyroid carcinoma for predicting central lymph node metastasis
Source: Front Oncol. 2023 Oct 30;13:1261080. doi: 10.3389/fonc.2023.1261080 (PMC10643192; doi:10.3389/fonc.2023.1261080)
Supplement: Supplementary file 1 [file DataSheet_1.docx]

**Supplementary Material**

**Supplementary Text 1: The specific principles of operation for patients with papillary thyroid carcinoma (PTC).**

All PTC patients in The Second Affiliated Hospital of Harbin Medical University, Harbin, Heilongjiang, China (training set, validation set and testing set) underwent comprehensive surgical treatment in accordance with the clinical TNM stage. Currently, lobectomy with isthmus or total/subtotal thyroidectomy plus central lymph node dissection and therapeutic ipsilateral lateral lymph node dissection was performed in clinically positive central lymph node metastasis or lateral lymph node metastasis patients in our institution. And prophylactic central lymph node dissection was performed and lateral lymph node dissection was not performed in cN0 papillary thyroid carcinoma patients (absence of any preoperative evidence of lymph node disease).

**Supplementary Text 2: The detailed ultrasound protocols.**

All preoperative ultrasound images were collected by two board-certified sonographers (Q.D. and Y.T., with 15 and 4 years of experience, respectively) using the same HITACHI HIVISION Avius (Hitachi Medical Corporation) equipped with a 5-13MHz linear probe. The ultrasound instrument was used to perform the same presetting criteria for thyroid ultrasound examination. The bilateral thyroid lobe, isthmus, and bilateral cervical lymph nodes was routinely performed.

Grayscale ultrasound images were adjusted to achieve optimal thyroid imaging effects. The tumor should be displayed at the center of the image to the extent possible, and subsequently the focus and time-gain compensation should be adjusted. During collection of color Doppler flow imaging (CDFI), the probe was minimally pressurized, and the color Doppler velocity scale (typically set to 1‒5 cm/s), color gain, and wall filtering were adjusted to avoid color noise and to show blood flow signals with optimal color. While obtaining strain elastography images, the probe was maintained perpendicular to the skin, and the size and position of the regions-of-interest (ROIs; these included normal glands around the nodules) were determined, with the focus placed to the center of the ROI. The probe scanning angle and amplitude were adjusted until the elastography image was stable. For all PTC cases, grayscale, CDFI, and strain elastography static images with typical sonogram characteristics in the maximum longitudinal and transverse sections were acquired. The multimodal ultrasound static images of the lesion were stored twice using the same method. Dynamic images were obtained by scanning the entire thyroid. Intra-observer consistency analyses were performed by Kappa.

**Supplementary Text 3: Specific segmentation ROI method.**

Images for subsequent processing were selected and segmented ROI by two sonographers (Q.D. and D.L., with more than 10 years of experience, respectively). They were unaware of whether each patient had central lymph node metastasis after surgery. When there was disagreement between the two sonographers, a consensus was reached through consultation. In all cases, the above two sonographers jointly confirmed and evaluated whether the ROI was satisfied. If the ROI was not satisfied, they needed to reoutline and reach a consensus.

When the ROI annotation for the grayscale and CDFI ultrasound images, the sonographers drew the ROI along the edge of the largest section of the tumor in the images as much as possible. If the edge of the lesion is blurred, the delineation region needs to contain the blurred edge. When the ROI annotation for the elastography images, the edge of the lesion was delineated on the grayscale image on the left side of the image in split-screen mode. To reduce the error, the ROI annotation of the lesion was then dragged to the elastography image area on the right side of the image to completely cover the real lesion area under the pseudo-color background of the elastography image. Here, the ROI in the multimodal ultrasound images delineated according to this method was used as a “mask” for feature extraction and model construction. The area of the ROI was equal to the area of the largest section of the tumor. In addition, the smallest rectangular box containing the edge of the lesion was only delineated as another ROI to localize the tumor and confirm the orientation of the “mask” region. The area of the ROI was slightly larger than that of the largest section of the tumor. Inter-observer consistency analyses were performed by Kappa.

**Supplementary Text 4:** **A brief description of three classifiers in this study.**

Adaptive boosting (AB) is a universal method use to synthesize strong classifiers after training multiple weak classifiers for the same training set, thus allowing the automatic selection of the most important features from high-dimensional data for preprocessing.

Linear discriminant analysis (LDA) is an effective classical dimensionality reduction method based on matrix pattern recognition with the objective of finding linear data transformations in the optimal discriminative subspace that increase the classification discriminative power.

Support vector machine (SVM) is one of the most commonly used classifiers in medical research and has good stability. It uses the kernel function to search for the optimal hyperplane in a high-dimensional space for the classification of samples.

**Supplementary Table 1: Intra-observers Kappa consistency test**

|  |  | Kappa coefficient | 95% CI |
| --- | --- | --- | --- |
| sonographer (Q.D.) |  |  |  |
| transversesection | grayscale | 0.823 | 0.645-0.941 |
|  | CDFI | 0.808 | 0.649-0.925 |
|  | elastography | 0.789 | 0.574-0.934 |
| longitudinal section | grayscale | 0.939 | 0.747-1.000 |
|  | CDFI | 0.831 | 0.683-0.949 |
|  | elastography | 0.773 | 0.607-0.902 |
| sonographer (Y.T.) |  |  |  |
| transverse section | grayscale | 0.860 | 0.627-0.920 |
|  | CDFI | 0.799 | 0.698-0.970 |
|  | elastography | 0.794 | 0.505-0.955 |
| longitudinal section | grayscale | 0.882 | 0.741-0.973 |
|  | CDFI | 0.818 | 0.655-0.931 |
|  | elastography | 0.744 | 0.416-0.946 |

**Supplementary Table 2: Inter-observers Kappa consistency test**

|  |  | Kappa coefficient | 95% CI |
| --- | --- | --- | --- |
| selected images (Q.D. and D.L.) |  |  |  |
| transverse section | grayscale | 0.946 | 0.854-1.000 |
|  | CDFI | 0.854 | 0.686-0.976 |
|  | elastography | 0.845 | 0.694-0.955 |
| longitudinal section | grayscale | 0.907 | 0.779-1.000. |
|  | CDFI | 0.874 | 0.743-0.969 |
|  | elastography | 0.835 | 0.661-0.955 |
| ROI satisfaction (Q.D. and D.L.) |  |  |  |
| transverse section | grayscale | 0.875 | 0.765-0.953 |
|  | CDFI | 0.775 | 0.635-0.894 |
|  | elastography | 0.816 | 0.693-0.914 |
| longitudinal section | grayscale | 0.868 | 0.720-0.973 |
|  | CDFI | 0.845 | 0.702-0.953 |
|  | elastography | 0.844 | 0.686-0.962 |
